# Design and Optimization of a Second-Generation Extruded Snack Using Carrot Waste, Blue Corn Flour, and Ellagic Acid as Functional Ingredients

**DOI:** 10.3390/foods14101657

**Published:** 2025-05-08

**Authors:** Yaír Adonaí Sánchez-Nuño, Karla Nuño, Alma Hortensia Martínez-Preciado, Jorge Manuel Silva-Jara, Carlos A. Velázquez-Carriles, Carlos Alberto Gomez-Aldapa, Angélica Villarruel-López

**Affiliations:** 1 Departamento de Farmacobiología, Centro Universitario de Ciencias Exactas e Ingenierías, Universidad de Guadalajara, Guadalajara 44430, Mexico; yair.sanchez@academicos.udg.mx (Y.A.S.-N.); jorge.silva@academicos.udg.mx (J.M.S.-J.); 2 Departamento de Psicología, Educación y Salud, Nutrición, Instituto Tecnológico de Estudios Superiores de Occidente, Tlaquepaque 45604, Mexico; karlanuno@iteso.mx; 3 Departamento de Ingeniería Química, Centro Universitario de Ciencias Exactas e Ingenierías, Universidad de Guadalajara, Guadalajara 44430, Mexico; alma.martinez@academicos.udg.mx; 4 Departamento de Ingeniería Biológica, Sintética y de Materiales, Centro Universitario de Tlajomulco, Universidad de Guadalajara, Carretera Tlajomulco, Santa Fé, Km 3.5, 595, Tlajomulco de Zúñiga 45641, Mexico; arnulfo.velazquez@academicos.udg.mx; 5 Área Académica de Química, Instituto de Ciencias Básicas e Ingeniería, Universidad Autónoma del Estado de Hidalgo, Mineral de la Reforma 42090, Mexico

**Keywords:** functional foods, blue maize, carrot bagasse, ellagic acid, extrusion, second generation snacks, food design

## Abstract

Blue maize is rich in bioactive compounds which are at risk of extinction due to monoculture practices. Carrot bagasse, considered a byproduct of the food industry, contains compounds that have been shown to benefit human health while also enhancing sustainability. Ellagic acid can prevent and assist in the treatment of various pathologies. Extrusion is a process characterized by its use of low energy, which minimizes the degradation of nutrients and bioactive compounds compared to other technologies. The objective of this research was to develop a functional food with high value of sensorial acceptance, desirable physicochemical, and antioxidant properties, using an 85:13:2% mixture of nixtamalized blue maize flour, carrot bagasse flour, and ellagic acid, processed with optimal conditions of extrusion determined with a surface response model. Operational conditions using a central rotatable experimental design were die temperature (DT = 120–170 °C), and screw speed (SS = 50–240 rpm), while response variables were physicochemical properties (expansion index, bulk density, breaking force, water absorption index and water solubility index) and antioxidant activity (free phenols inhibition of ABTS and DPPH). Sensory analysis, bromatological characterization and ellagic acid content determination with HPLC-DAD in reversed phase were also made. The optimal operational conditions were found to be DT = 144 °C and SS = 207 rpm, resulting in a mixture with high sensorial acceptability on a five-point hedonic scale. The optimized functional food may be used to promote the utilization of endemic ingredients and reduce food waste in the treatment of pathologies and prevention of diseases due to its high antioxidant activity attributed to phenolic and terpene compounds.

## 1. Introduction

The concept of functional food originated in Japan in the mid-1980s, when the Japanese government began providing financial assistance to research programs focused on the capacity of certain foods to influence physiological functions [1]. Nonetheless, to this day, a unique definition of functional food is non-existent.

Nowadays, the accepted definition of functional foods is “integral or whole foods, together with fortified foods, enriched or improved, that have a potentially beneficial effect on health when consumed as part of a varied diet in regular intervals at effective levels” [2,3]. These foods contain bioactive compounds that contribute to overall well-being, reducing disease risk. For this, a functional food must exhibit characteristics such as [4,5]: 1. They can be found in conventional foods with inherent sensorial characteristics [6]. It contains physiologically functional components not consumed at medicinal or therapeutic levels [7]. Impart physiological benefits scientifically proven when consumed as part of a regular diet, but not as isolates or pills [8]. Scientifically and clinically proven safety for long-term product consumption for the target population [6]. Contain functional components (either nutrients or phytochemicals) present in their natural form or added to the food [7]. It can be used for prevention and/or treatment of pathologies [8].

Functional and nutraceutical foods have been identified as one of the leading food categories in which research and development are focused [3,9]. The total global market value of functional foods in 2019 was $177.77 billion; it is estimated that by 2027, this value may rise to $267.92 billion, representing a 50.71% increase [10]. According to Hasler [11], functional food demand is expected to continue increasing in the years to come, as consumers have developed an interest in self-care, primarily due to the rising costs associated with aging. In the USA, in particular, the functional food market increased from $68,600 million to $92,210 million from 2018 to 2023, with an annual rate of 6.6% [10], while in Mexico, the increase from 2018 to 2019 was 5.4%, with sales around $6.7 million [12].

On the other hand, sustainable food production seeks to diminish organic waste generation (SDG 12: responsible production and consumption) [5]. In this sense, carrot bagasse (*Daucus carota* L.), a byproduct of the agri-food industry, has been a research focus due to its potential for improving sustainability and circular economics [13]. This residue, traditionally disposed of, contains bioactive compounds such as dietary fiber, antioxidants, and vitamins, which can be leveraged to produce functional foods and nutritional supplements [14]. Mexico is a significant producer of carrots, contributing substantially to the global market. In 2024, Mexico is projected to become the ninth-largest global agri-food producer. Carrots are among the various crops produced, with a considerable amount of byproducts generated during processing. According to the Agricultural Marketing Resource Center, fresh market carrots were harvested from 69,700 acres in 2020, yielding approximately 3.4 billion pounds. While specific data on carrot byproducts in Mexico are limited, the overall production figures suggest a substantial amount of waste that could be repurposed for sustainable practices [15].

The use of carrot bagasse in cereal elaboration for breakfasts rich in fiber through extrusion has improved the chemical characteristics and antioxidant capacity of these products [16]. Also, the transformation of carrot residues contributes to organic waste management and the reduction in dump loads [17]. The biorefinery of carrot byproducts enables the production of materials with added value, such as ethanol, carotenes used as antioxidants, and pectin, which serves as a natural thickener [18]. These applications not only optimize resource utilization but also create new market opportunities and encourage more sustainable agricultural practices.

Maize crops (*Zea mays* L.), including blue variety, are part of Latin American countries’ biological and cultural patrimony, including Mexico [19]. To this day, it can be classified into more than 250 species in the United States, with 59 of them found in Mexico [19]. Several varieties have been derived from these species, a result of the process of selection and improvement initiated by rural communities thousands of years ago, primarily in the Mesoamerican region. Blue maize constitutes a small portion of overall maize production in Mexico, making up approximately 0.3% of the 27.8 million metric tons of maize harvested. Despite its limited production, blue maize is cultivated by smallholder farmers who have preserved native varieties for generations [20]. The demand for blue maize has grown, particularly in high-end restaurants and supermarkets in Mexico City and other regional cities. Blue maize products, such as tortillas and chips, are increasingly popular due to their unique flavor and health benefits, including antioxidants that may lower risks for coronary disease, diabetes, arthritis, and cancer [21].

Blue maize phytochemicals have received less attention than those found in fruits, vegetables, and other cereals, including those from other maize species. Blue maize contains an elevated quantity of antioxidant compounds, such as anthocyanins and anthocyanidins, primarily cyanidins, as well as a higher protein content than white maize, which reduces the glycemic index. Since blue maize is cultivated in an agroecological manner, it often produces less waste and requires a minimal number of pesticides and herbicides, or sometimes none. Additionally, nixtamalization has been shown to enhance protein quality and bioavailability, release antioxidant compounds (such as anthocyanins, ferulic acid, and anthocyanidins), inhibit the formation of aflatoxins, and increase niacin (vitamin B3) availability [22,23]. The consumption of blue maize and its derivatives and byproducts has been linked to a reduction in chronic disease risks, including cardiovascular diseases, type 2 diabetes, and certain types of cancer, as well as improvements in digestive tract health due to its anthocyanin content [19,24].

Ellagic acid is found in fruits such as pomegranates, persimmons, raspberries, black raspberries, strawberries, peaches, and plums, as well as in seeds like walnuts and almonds, and certain vegetables. It can be found free or bound to other compounds, mostly with complex polymers called ellagitannins, which can be hydrolyzed at physiological pH and by intestinal microbiota, increasing its plasma levels after the intake of fruits and vegetables [24]. This acid is one of the primary antioxidants, along with ascorbic acid and α-tocopherol. Its intrinsic antioxidant properties have been attributed to its ability to eliminate free radicals, like essential vitamins. The presence of four hydroxyl groups and two lactones enables ellagic acid to eradicate a wide range of reactive oxygen species (ROS) and nitrogen reactive species [25]. Several studies have highlighted the potential of ellagic acid as a candidate for treating and preventing diseases and chronic inflammatory conditions, including type 2 diabetes mellitus, cardiometabolic diseases, and various types of cancer [26,27,28].

In this regard, dough and products with starch extrusion, such as those made from cereals, have been widely used in the food industry to produce snacks [29,30]. The process of extrusion enables the production of low-fat snacks. It promotes the formation of resistant starch, which contains fewer calories and contributes energetic substrates to the intestinal microbiota [31]. As a result, extrusion has become more popular for the snacks generation with adequate nutritional content [32]. On the other hand, the food industry generates higher quantities of waste that are usually discarded in dumps and allowed to decompose [33]. Including this waste in technological processes can add value to the industry and reduce contamination related to these wastes, reducing the impact on ecosystems [34].

The scientific field of extruded functional foods has seen significant advancements in recent years. Extrusion technology, which involves high-temperature, short-time processing, has been utilized to enhance the nutritional and functional properties of various food products. Researchers have focused on incorporating bioactive compounds, proteins, and dietary fibers into extruded foods to improve their health benefits. For instance, studies have shown that adding ingredients like anthocyanins, carotenoids, and phenolic compounds can increase the antioxidant capacity and overall nutritional value of extruded snacks [35]. This technology has also been applied to cereals, legumes, and pulses, resulting in products that are not only nutritious but also have improved sensory qualities [36].

The benefits of extruded functional foods for nutrition are substantial. Extrusion can retain essential nutrients and improve the digestibility of food materials, making them more accessible to the body [35]. This process can also reduce the presence of anti-nutritional factors, which can interfere with nutrient absorption [37]. By enhancing the nutritional profile of foods, extrusion technology helps in addressing dietary deficiencies and promoting overall health. For example, extruded snacks enriched with protein and fiber have been shown to support muscle growth and digestive health [36]. Additionally, the incorporation of bioactive compounds can provide specific health benefits, such as reducing oxidative stress and inflammation. Extruded functional foods also play a crucial role in disease prevention. The inclusion of bioactive compounds like polyphenols, flavonoids, and antioxidants in extruded products can help in preventing chronic diseases such as cardiovascular diseases, diabetes, and cancer [38]. These compounds have been shown to reduce the risk of developing these conditions by combating oxidative stress and improving metabolic health [39]. Furthermore, extruded foods can be designed to target specific health issues, such as weight management and gut health, by incorporating ingredients that promote satiety and support the growth of beneficial gut bacteria [40]. Overall, the advancements in extrusion technology and the development of functional foods offer promising solutions for improving nutrition and preventing diseases.

This research aimed to design and develop a functional food prospect composed of blue maize (*Zea mays* L.), carrot bagasse (*Daucus carota* L.), and ellagic acid through extrusion, contributing to the design and development of nutritious alternatives to conventional snacks, as well as promoting the use of food waste and fast, low-energy processing methods.

## 2. Materials and Methods

### 2.1. Raw Materials

Blue maize nixtamalized dough (*Zea mays* L.) was obtained from a local mill specializing in organic and agroecological blue maize from the State of Mexico, located in Zapopan, Jalisco, Mexico. The chemical composition of nixtamalized blue maize dough includes various components such as starch, protein, and calcium, which are influenced by the nixtamalization process [41,42]. The carrot bagasse was obtained from several natural juice establishments in Guadalajara, Jalisco, Mexico, where it was collected immediately after juice extraction to minimize exposure to air and the proliferation of microorganisms. Then, it was transported in hermetically sealed and refrigerated containers to maintain a temperature of 4 °C. For storage, a refrigerated environment at 4 °C and a relative humidity of 85–90% were established, using plastic or stainless-steel containers with hermetic seals. These containers were washed and disinfected with chlorine at a concentration of 100 ppm before use. Carrot bagasse, a by-product of carrot processing, contains significant amounts of dietary fiber, vitamins, and minerals [43]. Ellagic acid (90%) was purchased commercially from Pure Bulk Inc. (Roseburg, OR, USA).

### 2.2. Dough Obtaining

Blue maize nixtamalized dough and carrot bagasse were dehydrated at 60 °C for 8 h, and 60 °C for 12 h, respectively, in a forced air circulation stove (Thermolyne Oven Series 9000, Emeryville, CA, USA); then, they were ground in a blender (Oster, BPST02-B, Cleveland, OH, USA) and sieved in US 30 mesh to obtain particle sizes smaller than 595 µm. The mixture was composed of 85% blue maize dough, 13% carrot bagasse, and 2% ellagic acid. The proportion of the ingredients was defined based on the group’s experience [32,44,45,46,47].

### 2.3. Extrusion Process

The humidity level of the flour mixture was adjusted to 20% with purified water. Then, samples were processed in an extruder with one screw (Brabender Instruments Inc., model 20DN/8-235-00, Duisburg, CW, Germany), at a feed rate of 30 g/min. The moisture and feed rate were set at the values mentioned above due to previous studies [32,44,45,46,47]. The cylindrical extruder is divided into three heating zones; the first two were fixed with negative gradients of 15 °C, corresponding to the experimental design of the third zone, which encompassed the matrix and exit die. The speed of screw was variated in accordance with the experimental design, using an exit diameter of 3 mm, and a screw with a compression relation of 3:1 (Table 1).

### 2.4. Physicochemical Characterization of Extruded Products

#### 2.4.1. Expansion Index (EI)

The expansion index (EI) was calculated by dividing the diameter of the extruded by the diameter of the exit die. 50 determinations were made for each treatment.

#### 2.4.2. Apparent Density or Bulk Density (BD)

The apparent density (g/cm^3^) was determined by dividing the weight of the extruded piece by its apparent volume (cm^3^). The apparent volume was determined using Equation (1).

(1) $$V=\frac{1}{4}\left(\pi \times d^{2}\times h\right)$$

where *d* (m) is the diameter of the extruded product and *h* (m) is the average length.

#### 2.4.3. Hardness or Breaking Force (BF)

The breaking force (BF) to cut the expanded product was measured with a texture analyzer (TA-TX2, Stable Micro Systems, Ltd., Godalming, Surrey, UK). Extruded samples were placed horizontally on a platform, and a plain knife with a 1 mm thickness was used. The probe descent rate was 2 mm/s, and the maximum breaking distance was 6 mm. Thirty measurements were made for each treatment, and the results were expressed in Newtons (N).

#### 2.4.4. Water Absorption Index (WAI) and Water Solubility Index (WSI)

The water absorption index and water solubility index were determined according to Navarro-Cortez et al. [32], modifying the weight from 1 to 3 g.

#### 2.4.5. Free and Bound Phenols with ABTS and DPPH

Methanolic extracts from crude and extruded samples were obtained to determine the radical-scavenging activity using the ABTS (2,2′-azino-bis(3-ethylbenzothiazoline-6-sulfonic acid)) and DPPH (2,2-diphenyl-1-picrylhydrazyl) methods, as described by Jinli Zhang et al. [48] for free phenols (FPI) and bound phenols (BPI). Each sample was measured in triplicate, and the average was used for the experimental design. The results for each radical were expressed as a percentage of inhibition, estimated with Equation (2):

(2) $$Inhibition\;\left(\%\right)=\frac{A_{blank}-A_{sample}}{A_{blank}}\times 100$$

where *A_blank_* and *A_sample_* are the absorption of the blank and sample, respectively.

### 2.5. Second-Generation Snack Optimization

#### 2.5.1. Experimental Design

A central rotatable experimental design with two independent variables was used as follows: Variables were die temperature and screw speed, with five levels and thirteen experiments over nine response variables (expansion index (EI), apparent density (BD), breaking force (BF), water absorption index (WAI), water solubility index (WSI), antioxidant activity of free and bound phenols with ABTS and DPPH).

#### 2.5.2. Sensory Analysis

Two sensory tests were conducted with 34 untrained students and workers from the Centro Universitario de Ciencias Exactas e Ingenierías (CUCEI) of the Universidad de Guadalajara (UDG), comprising 17 women and 17 men, aged between 18 and 70 years, who typically consume snacks. Tests were divided into two categories: discriminatory-descriptive sensorial tests and a 5-point hedonic scale, both presented as a Likert-type survey. Panelists evaluated four extruded snacks with different die temperatures and screw speed to determine which combination was the most accepted considering a scale from 1 to 4, where 1 was “bad” and 4 “excellent” [49]. The sample with the highest scores was further evaluated for color, texture, flavor, and acceptability using a hedonic scale of 5 points (1 = ‘hated it’, 2 = ‘did not like’, 3 = ‘indifferent’, 4 = ‘liked’, 5 = ‘loved it’) [50]. Panelists washed their mouths after tasting each sample.

#### 2.5.3. Bromatology Analysis

The bromatological analysis of the extruded snacks produced with the optimum conditions (die temperature 144 °C, screw speed 207 rpm), according to the mathematical model, to determine the nutrimental composition of the food. The analyses were performed according to Mexican norms, and they included humidity (NMX-F-227-1982), ashes (NMX-FRO66-S-1978), protein (NMX-F608-NORMEX-2002), total, saturated and unsaturated fats (NMX-F-089-S-1978), dietary fiber (NOM-086-SSA1-1994), total carbohydrates (NOM-051-SCFI/SSAI-2010), total reducing sugars (NMX-F-312-NORMEX-2016), energetic content (NOM-051-SCFI/SSAI-2010), and sodium (NMX-F-360-S-1981). All analyses were duplicated, and results were expressed as the average.

#### 2.5.4. Ellagic Acid Content in the Final Product

An HPLC (Agilent Technologies 1260 Infinity 1200 series, Agilent, Santa Clara, CA, USA) equipped with a diode arrange detector was used. The separation was conducted on a reverse-phase column (Hypersil ODS, Thermo Fisher Scientific: Waltham, MA, USA, 4.6 × 150 mm, 5 µm). The compounds were identified at a wavelength of 253 nm and a temperature of 40 °C, with an injection volume of 10 µL. For the quantification of ellagic acid, a standard curve was constructed using a standard of 90% purity (PureBulk, Inc., Roseburg, OR 97471, USA) in volumes ranging from 10 to 50 µL. Each point was measured in triplicate to ensure precision and reproducibility. The mobile phase was acidified with 0.7% phosphoric acid in a 40:60 methanol-water mixture to optimize the separation of analytes. The elution concentration was 25 mg of the sample in 5 mL of the mobile phase, with 300 µL of 0.1 N NaOH. The samples of the extruded snacks were prepared and diluted by injecting 10 µL under the conditions previously mentioned in the HPLC. Data obtained from DAD were used to determine the concentration of ellagic acid in samples, which was compared to the standard curve.

### 2.6. Statistical Analysis

The data were analyzed and adjusted to a second-order regression model to obtain the regression coefficients. Graphs of surface response were obtained using the statistical package Design-Expert 13.0.5.0 (Stat-Ease, Inc., Minneapolis, MN, USA). The importance of each term in the equation was further analyzed using an analysis of variance (ANOVA) for each variable. The model for surface response with linear, quadratic and interaction terms were used to relate each variable with the extrusion die temperature and screw rotation speed.

$$y=\beta_{0}+\beta_{1}x_{1}+\beta_{2}x_{2}+\beta_{3}x_{3}+\beta_{12}x_{1}x_{2}+\beta_{13}x_{1}x_{3}+\beta_{23}x_{2}x_{3}+\beta_{11}x_{11}+\beta_{22}x_{22}+\beta_{33}x_{33}$$

## 3. Results

**Figure 1:**
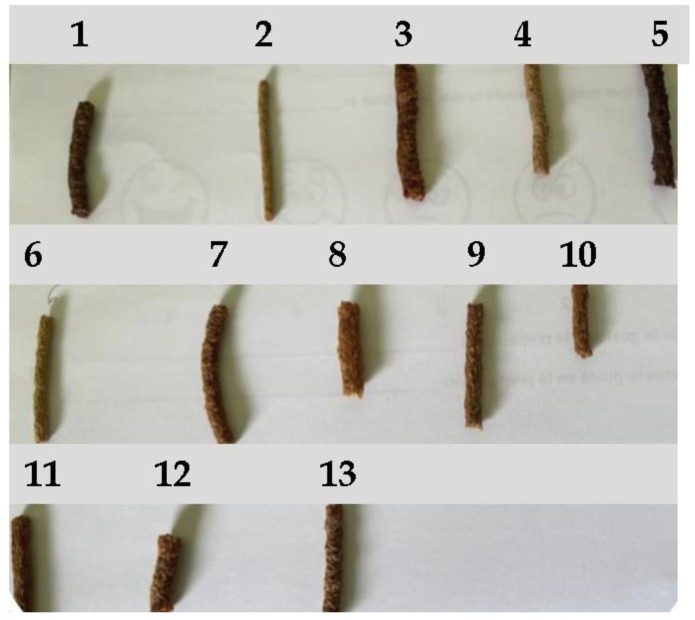
Appearance of each extruded snack obtained for the determination of optimal processing conditions, with variable die temperatures (120–170 °C) and screw speed (50–240 rpm). Numbers represent each run.

The appearance of extruded snacks in the 13 runs to determine optimal die temperature and screw speed is shown in Figure 1.

The parameters measured for each sample showed an R^2^ value of ≥0.7 and an adequate precision (AP) of ≥4 (Table 2); thus, they were adjusted to the model, which included the expansion index, apparent density, breaking force, water absorption index, ABTS inhibition for free phenols, and DPPH inhibition for free phenols. These parameters were considered for the superposition of areas and the obtaining of optimal conditions for the formulation process. The other parameters, water solubility index, inhibition of bound phenols by ABTS, and DPPH, were not considered for the analysis.

### 3.1. Regression Coefficients and ANOVA

The regression coefficients for the analyzed responses are presented in Table 3. Both factors, die temperature (DT) and screw speed (SS), showed a significant effect (*p* < 0.05) in their linear (β_1_) or quadratic (β_11_) terms on most of the responses studied, except for DPPH free phenol inhibition (DFPI). Furthermore, for BF and WAI, the reduced cubic model (β_22_ and β_22_β_1_) also had a significant effect. The interaction terms of the models (Table 3) were generally significant for two responses. At the same time, the linear coefficients were significant in four responses, as well as the quadratic coefficients in five responses. Additionally, Table 2 shows the analysis of variance (ANOVA) for the analyzed response variables. The models were accurate for all responses, with values of R^2^ > 0.77, *p* of *F* (model) < 0.05 in four responses, and variability coefficient (CV) < 16% (except for BF and DFPI). However, all responses showed an adequate precision (>5.5). Only two responses presented a significant lack of fit (BD and BF).

**Table 2:** Variance analysis for analyzed responses.

Response	*R* ^2^	Adequate Precision	CV (%)	*F* Value	*p* of *F* _(model)_	Lack of Fit
EI	0.9057	11.3216	7.62	13.44	**0.0018**	0.3526
BD (AD)	0.8282	8.4050	15.47	6.75	**0.0132**	0.0298
BF (H)	0.9270	12.7868	26.30	17.79	**0.0007**	0.0015
WAI	0.8297	6.5469	7.14	3.48	0.0944	0.5344
AFPI	0.8123	9.7396	15.00	6.06	**0.0176**	0.2124
DFPI	0.7754	5.6930	23.99	2.47	0.1688	0.7033

Note: CV, coefficient of variation; EI, expansion index; BD (AD), bulk density (apparent density); BF (H), breaking force (hardness); WAI, water absorption index; AFPI, ABTS-free phenols inhibition; DFPI, DPPH-free phenols inhibition. Numbers in bold refer to statistically significant *p*-values.

**Table 3:** Regression coefficients of the models and significance levels for analyzed responses.

Coefficients								
Response	Intercept	Linear		Quadratic		Interaction		
		A	B	AB	A^2^	B^2^	A^2^B	AB^2^
EI	+1.96	−0.41 ***	+0.36 **	+0.03	+0.29 *	+0.006	+0.43	+0.14
BD (AD)	+0.30	−0.02	−0.13 **	+0.06	+0.09	+0.04	−0.16	−0.15
BF (H)	+13.21	−17.27 ***	−5.48 *	+10.86 *	+15.31 **	−1.69	−13.03	+15.9 *
WAI	+5.72	+0.09	−0.80 *	−1.23 *	−0.22	−0.13	+2.72 *	−0.99
AFPI	+48.60	+5.72	+6.66	−3.66	−17.52 *	+18.69 *	−26.55	+15.99
DFPI	+14.22	−0.91	+4.22	+0.37	−5.99	+3.16	−16	+8.29

Note: EI, expansion index; BD (AD), bulk density (apparent density); BF (H), breaking force (hardness); WAI, water absorption index; AFPI, ABTS-free phenols inhibition; DFPI, DPPH-free phenols inhibition; A, B, …, AB2, regression coefficients. A: die temperature; B: screw speed. Numbers in bold refer to statistically significant *p*-values. * *p* < 0.05; ** *p* < 0.01; *** *p* < 0.001.

### 3.2. Expansion Index

**Figure 2:**
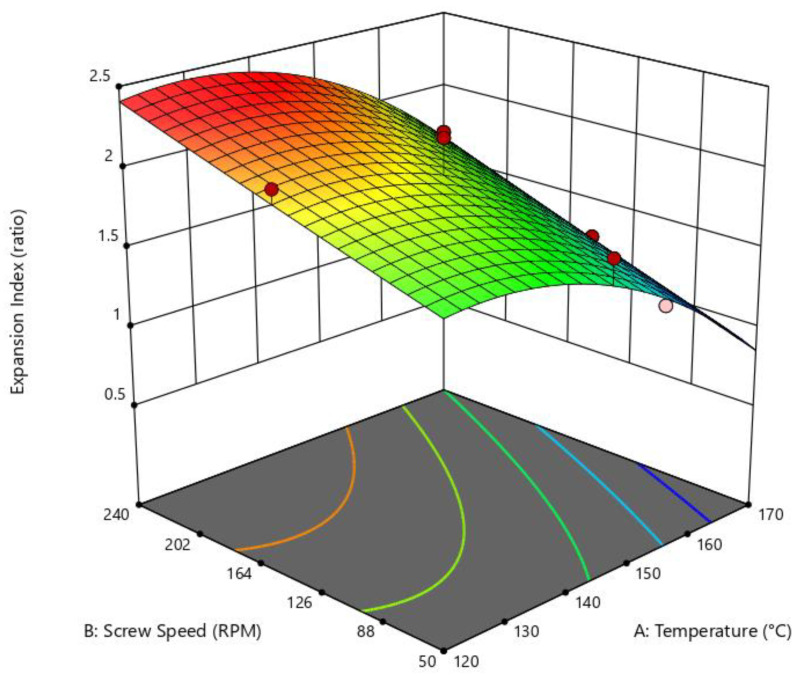
Surface response of the effect of die temperature and screw speed on expansion index. Quadratic model: *p* = 0.0018; R^2^ = 0.9057; adeq. Precision = 11.3216.

The effect of the die temperature and screw speed on the expansion index were analyzed; these results presented a statistically significance of *p* = 0.0018, R^2^ = 0.9057, and an adequated precision of 11.32 in the quadractic model of the ANOVA test. Figure 2 shows the inference of the independent variables “die temperature” and “screw speed” on the “expansion index” variable, where high SS and low die temperature favored a major EI of the extruded snacks. In contrast, low SS and high die temperature had an inverse effect.

### 3.3. Apparent Density

**Figure 3:**
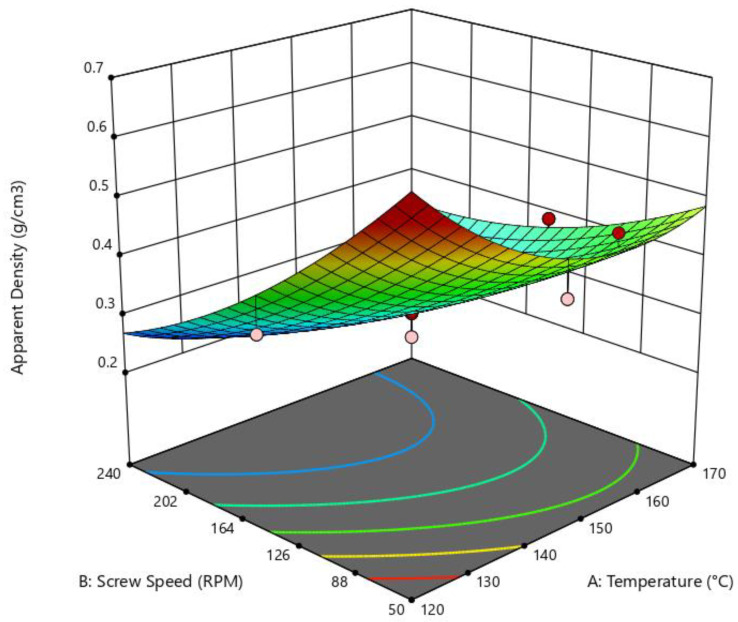
Surface response of the effect of die temperature and screw speed on appararent density. Quadratic model: *p* = 0.0132; R^2^ = 0.8282; adeq. Precision = 8.4050.

In this study, the effect of die temperature and SS on apparent density was determined, with results proving to be significant (*p* = 0.0132, R^2^ = 0.8282, adequate precision = 8.4), as indicated by the quadratic model of the ANOVA test. The effect of the independent variables on apparent density is illustrated in Figure 3, where it can be observed that low SS results in a higher density. In contrast, for higher SS, the density of the extruded snack is inferior.

### 3.4. Breaking Force

**Figure 4:**
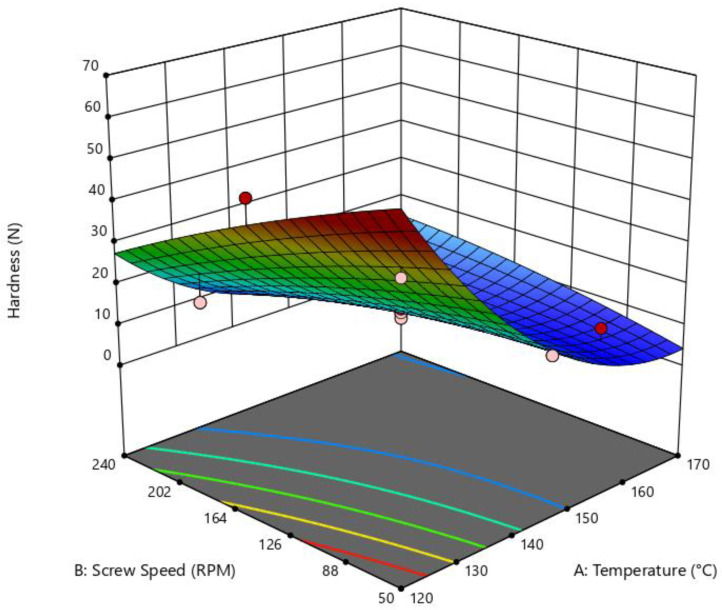
Surface response of the effect of die temperature and screw speed on breaking force. Quadratic model: *p* = 0.0007; R^2^ = 0.9270; adeq. Precision = 12.7868.

The influence of the independent variables on the breaking force had a significant impact (*p* = 0.0007, R^2^ = 0.927, adequate precision = 12.79) according to the quadratic model of the ANOVA test. Figure 4 illustrates the relationship between die temperature and SS on BF, showing that snacks processed at low SS and die temperature exhibited higher hardness (measured in N). In contrast, higher values of independent variables diminish it.

### 3.5. Water Absorption Index

**Figure 5:**
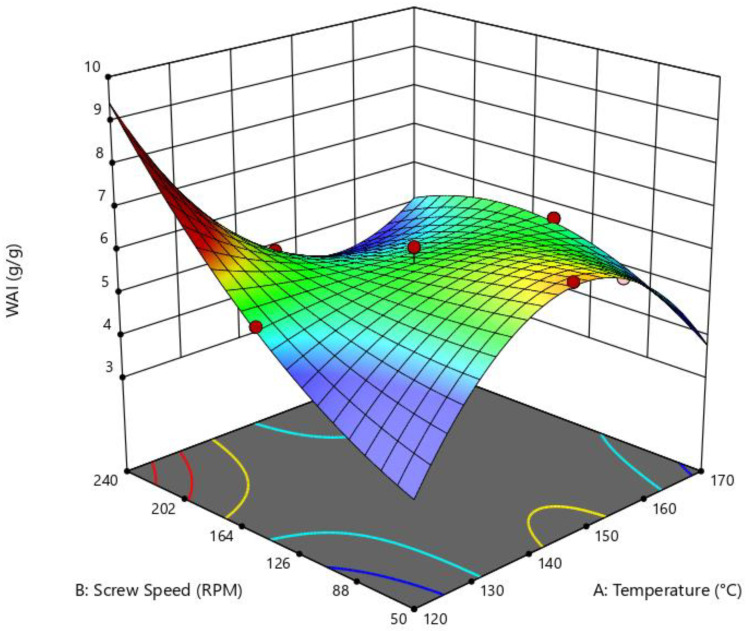
Surface response of the effect of die temperature and screw speed on water absorption index. Reduced cubic model: *p* = 0.0944; R^2^ = 0.8297; adeq. Precision = 6.5469.

For water absorption index (WAI), the effect of die temperature and screw speed were not significant (*p* = 0.0944, R^2^ = 0.8297, adequate precision = 6.55); nevertheless, they had a meaningful tendency in the reduced cubic model of the ANOVA test, for which a significant relation between die temperature and screew speed on WAI does not exists. High values of WAI (g/g) can be achieved when the extruded snack is treated at high SS and low die temperatures, as shown in Figure 5.

### 3.6. Free Phenols Inhibition of ABTS

**Figure 6:**
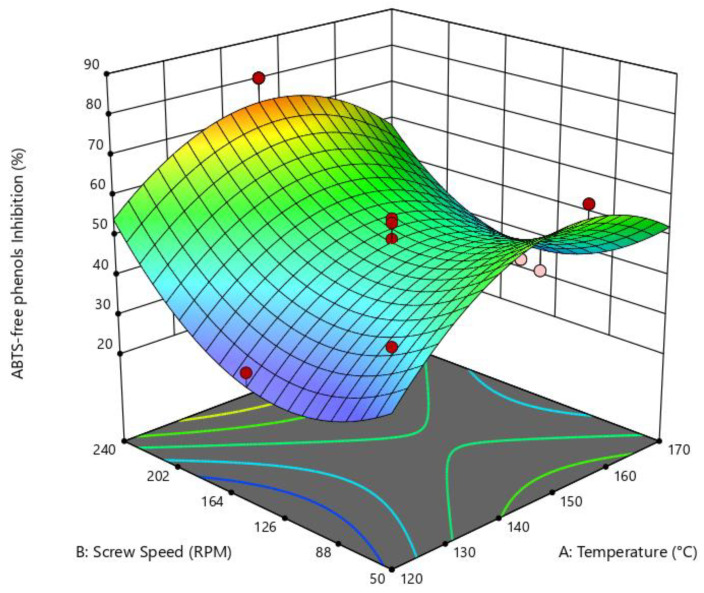
Surface response of the effect of die temperature and screw speed on ABTS inhibition with free phenols. Quadratic model: *p* = 0.0176; R^2^ = 0.8123; adeq. Precision = 9.7396.

The free phenols inhibition of the ABTS radical (AFPI) were statistically significant for die temperature and SS, in the quadratic model (*p* = 0.0176, R^2^ = 0.8123, adecuate precision = 9.74), according to the Design-Expert program, finding a relation between die temperature and SS, and AFPI, with good adjustment to the model. In Figure 6, it can be seen that high SS and medium die temperature (140–150 °C) resulted in higher inhibition of the ABTS radical (%) compared to free phenols.

### 3.7. Free Phenols Inhibition of DPPH

**Figure 7:**
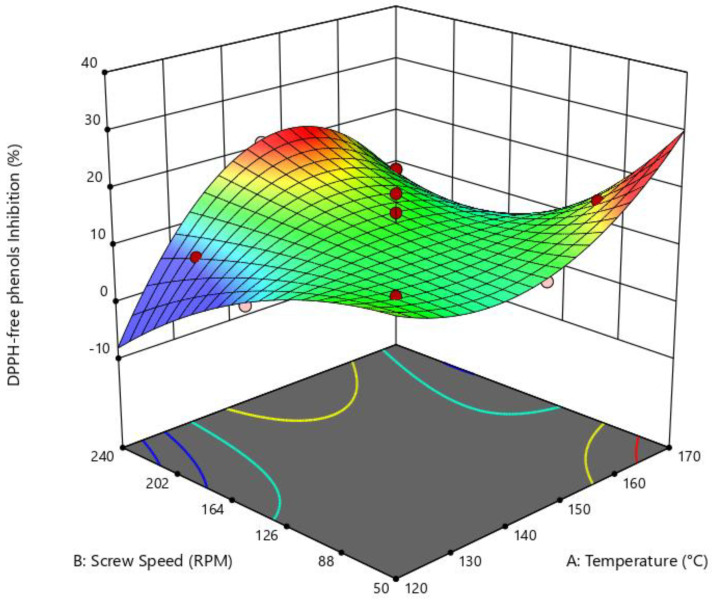
Surface response of the effect of die temperature and screw speed on DPPH inhibition with free phenols. Reduced cubic model: *p* = 0.1688; R^2^ = 0.7754; adeq. Precision = 5.6930.

A reduced cubic model (Design-Expert) was used to determine the influence of die temperature and SS on the inhibition of DPPH with free phenols (DFPI). No significant difference was found, with a *p*-value of 0.1688, an R-squared value of 0.7754, and an adequate precision of 5.7; however, a good fit was determined. For this variable, both high and low SS (50 and 240 rpm) and medium-high die temperatures (140–160 °C) resulted in higher inhibition of the DPPH radical (%) (Figure 7).

### 3.8. Optimum Design of Extruded Snacks

**Figure 8:**
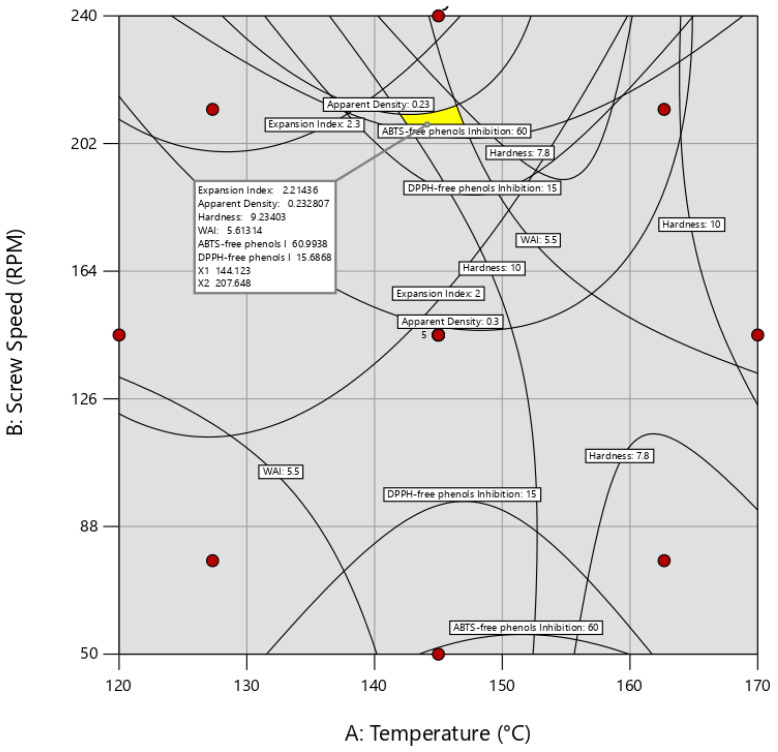
Area superposition of EI, BD, BF, WAI, WSI, AFPI, ABPI, DFPI, and DBPI regarding die temperature and screw speed in extrusion of snacks. Optimal operational conditions were established as 144 °C for die temperature and 207 rpm for screw speed.

Figure 8 depicts the superposition of areas of response variables regarding die temperature and screw speed. Optimal responses were established within the next optimal ranges: expansion index (EI) ranged from 2.00 to 2.30 (ratio), bulk density (BD) from 0.23 to 0.30 g/cm^3^, and breaking force (BF) from 7.80 to 10.00 N. Additionally, water absorption index (WAI) ranged between 5.50 and 6.70 g/g, ABTS-Free Phenols Inhibition (AFPI) from 60.00% to 80.00%, and DPPH-Free Phenols Inhibition (DFPI) from 15% to 21%. It should be noted that only response variables with R^2^ > 0.7 and an adequate precision > 4 were selected, which were EI, BD, BFPF, WAI, AFPI, and DFPI, while water solubility index (WSI), ABTS (ABPI), and DPPH (DBPI) inhibition with bounded phenols were excluded. According to the results, the optimum operational conditions were 207 rpm for screw speed, and 144 °C for die temperature. A Q-Q plot, residual analysis, ANOVA, and multiple regression analysis were conducted to validate the model’s predictability and fit mathematically.

### 3.9. Sensory Analysis

**Figure 9:**
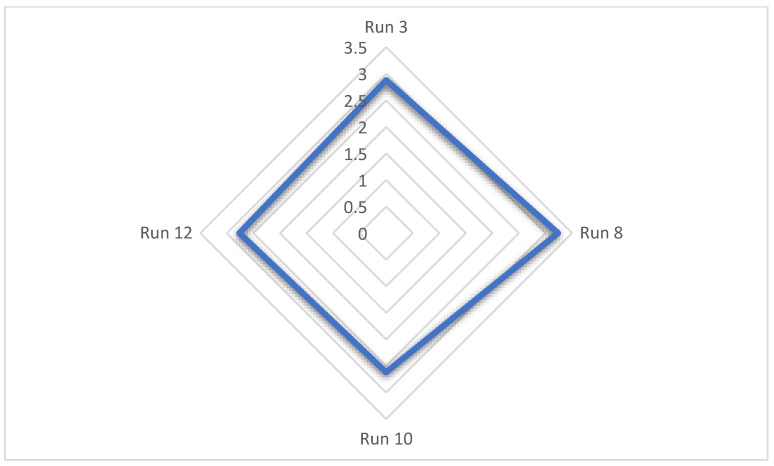
Radar chart of discriminatory-descriptive sensorial test of samples 3, 8, 10, and 12. Qualitative parameters were evaluated on a scale of 1 to 4, with scores ranging from “good” to “excellent”; Sample 8 was the most highly accepted. Sample 3 = 2.882, Sample 8 = 3.235, Sample 10 = 2.617; Sample 12 = 2.764. n = 34.

Discriminatory-descriptive sensorial tests on samples 3, 8, 10, and 12 are depicted in Figure 9, where an average score of 3.235 (*n* = 34) was found, corresponding to an intermediate level between “good” and “excellent”, indicating high acceptability of the product. Sensory analyses were only performed on samples 3, 8, 10, and 12; the other samples (1, 2, 4, 5, 6, 7, 9, 11, and 13) were inedible due to an unpleasant smell and taste.

**Figure 10:**
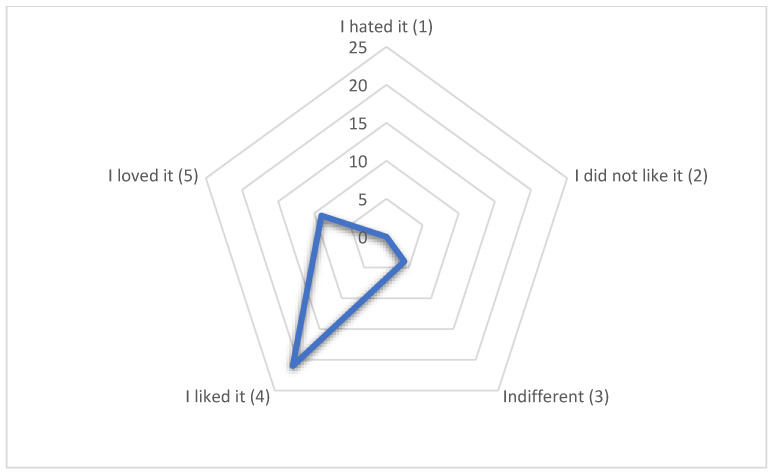
Radar chart of 5-point hedonic scale of Sample 8. Most participants concentrated in scale 4 (liked it), followed by 5 (loved it) and 3 (indifferent) (n = 34), demonstrating high acceptability. I hated it = 0%; I did not like it = 0%; Indifferent = 11.76%; I liked it = 61.76%; I loved it = 26.48%. Total score = 4.15 (between “I liked it” and “I loved it”).

Further tests with a 5-point hedonic scale (Figure 10) punctuated Sample 8 with more responses for “liked it” (scale 4), followed by “loved it” (scale 5), and last “indifferent” (scale 3), from a total of 34 evaluations, which showed high acceptability (61.76% for “liked it”). Only 11.77% of the panelists selected “indifferent”, while no one chose 1 or 0 (“did not like it” and “hated it”, respectively). These results regarding sensorial and organoleptic characteristics highlight the acceptability of the product, suggesting high commercialization levels. Interestingly, the descriptions of panelists mentioned that the least liked parameter was the end flavor, described as “acidity”. In contrast, the crunchy and expanded texture, as well as the initial taste, were the most liked parameters.

### 3.10. Bromatological Characterization of Final Product

The nutritional content of the extruded product is presented in Table 4, where the nutritional information labeling was constructed by the Mexican Official Norm NOM-051-SCFI/SSA1-2010. Humidity, ashes, protein, total fats, saturated fats, unsaturated fats, dietary fiber, total carbohydrates, total reducing sugars, energetic content, and sodium were quantified in duplicate.

### 3.11. Ellagic Acid Content Determination with HPLC-DAD in Reversed Phase

**Figure 11:**
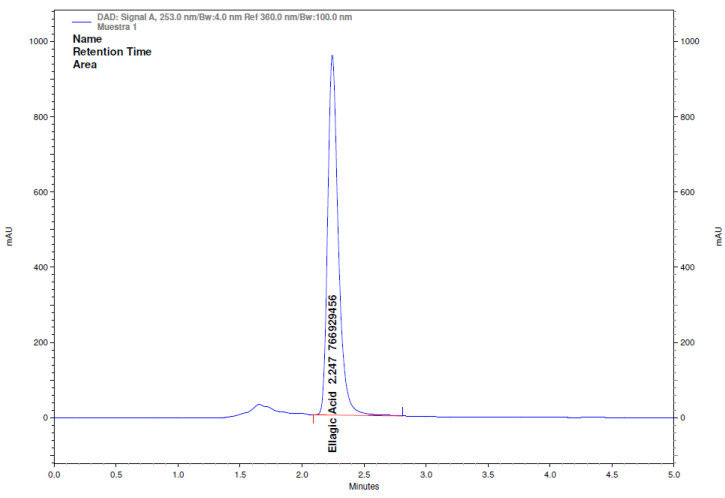
Chromatogram of ellagic acid detection in extruded snacks, obtained at optimal operational conditions, with integration of the area under the curve.

The ellagic acid content in the extruded snacks was measured using HPLC-DAD (Figure 11) to determine the loss and retained percentage. The test was performed in duplicate, and analysis showed a loss of 50.5% of ellagic acid. The ellagic acid content before the extrusion process was 1800 mg per 100 g of initial product (2000 mg of ellagic acid at 90% purity was used), while after the extrusion process, 891 mg remained per 100 g of extrudates.

### 3.12. Appearance of Extruded Snacks

**Figure 12:**
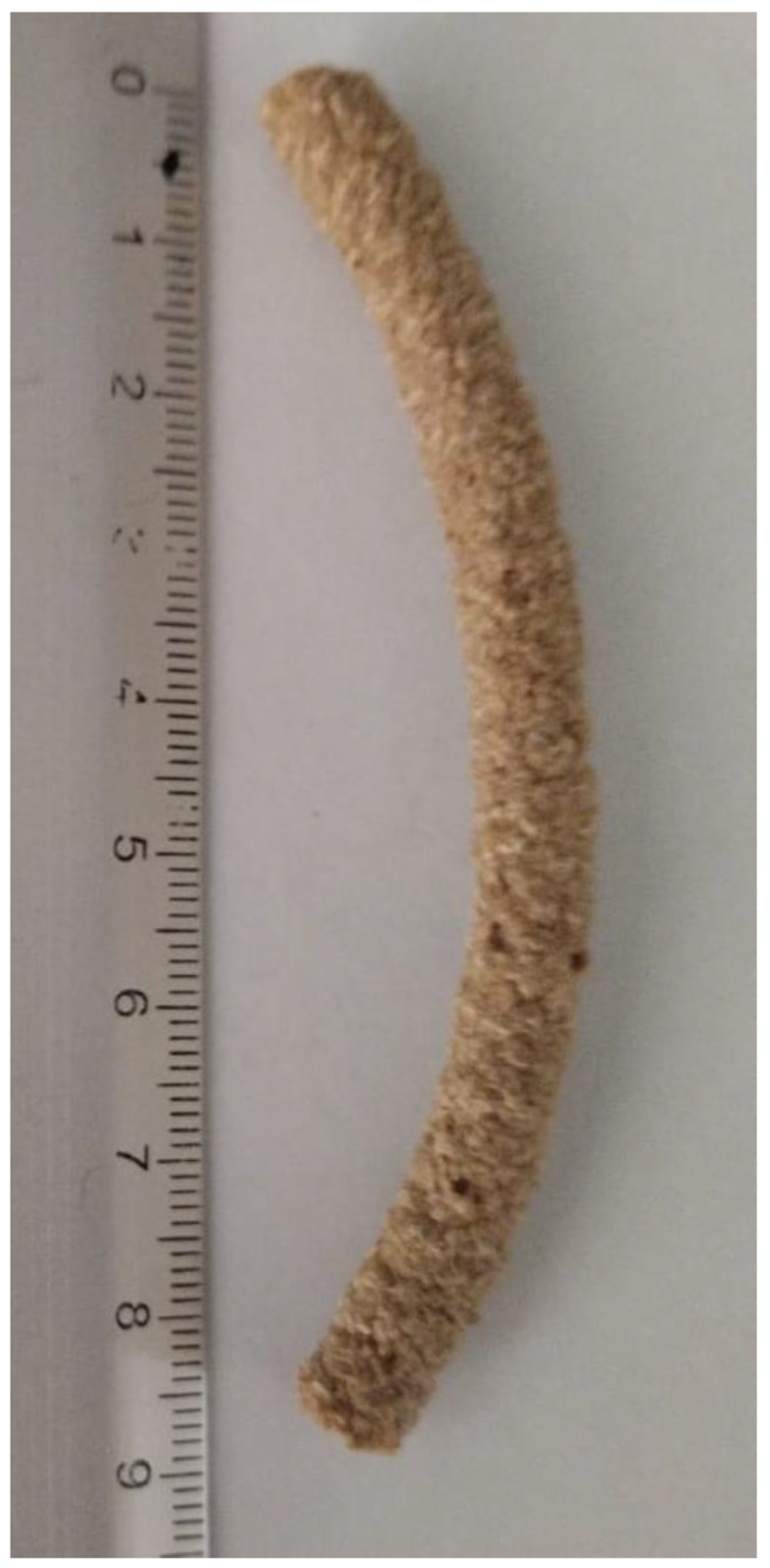
The final extrudate was obtained at optimal processing conditions (144 °C and 207 rpm).

Figure 12 depicts the appearance of the extruded snack obtained at optimal conditions of die temperature (144 °C) and screw speed (207 rpm).

## 4. Discussion

The utilization of food residues and waste, such as carrot bagasse, presents a significant opportunity for sustainable development and innovation in the food industry, particularly in the design and fabrication of functional foods aimed at enhancing consumer health and preventing various pathological conditions. Ellagic acid and bioactive compounds, such as anthocyanins and carotenoids, found in blue maize and carrots, respectively, have been shown to reduce the incidence of diseases like cardiovascular disease, metabolic syndrome, and type 2 diabetes mellitus, which are among the leading causes of mortality worldwide.

The integration of byproducts, such as carrot bagasse, in the formulation of these foods not only contributes to waste reduction but also offers nutritional and functional benefits, responding to the growing demand for innovative and healthy food. To date, no commercially available food products incorporating nixtamalized blue maize flour, carrot bagasse, and ellagic acid have been identified. This product contributes to the revalorization and reutilization of carrot bagasse, aligning with the Sustainable Development Goals (SDG) and incorporating agroecological, organic, and traditionally cultivated blue maize.

The significance of linear and quadratic regression coefficients suggest that die temperature and screw speed had a considerable impact on the response variables of the extruded snacks [51]. This is consistent with previous studies that demonstrated the influence of these parameters on various extrusion processes. For example, Sahu et al. [52] found that temperature has a significant impact on the quality of the final product in polymer extrusion.

The variance analysis (ANOVA) confirms the precision of the models applied, indicating that the models adequately describe the relationship between the independent and response variables. The high adequate precision (>5.5) and R^2^ values above 0.77 reinforce the validity of the models. However, the lack of significant adjustment of BD and BF suggests that other factors not considered may have had a considerable impact. The results of this study provide a solid base for future research and optimization of processes applied in the industry.

The expansion index, which measures the increase in volume of food during the extrusion process, is crucial for evaluating the texture and quality of the final product, as a higher index generally results in a lighter and crunchier product. This parameter is critical during snacks and cereals production; in general, a high EI is associated with high temperature and screw speed in extrusion. Therefore, it is unusual that extruded products exhibit lower EI when processed at high temperature and low screw speed. Nonetheless, this could be attributed to several factors: (1) The composition of raw materials; if it is not adequate, EI may be low even at high temperatures and screw speeds [53]. The chemical composition, such as starch, protein, fiber, and so on, has a non-negligible impact on the EI. For instance, starch processing involves significant energy consumption and emissions, contributing to the overall environmental impact [54]. Similarly, the type and source of protein can significantly affect environmental sustainability, with animal-based proteins generally having a higher carbon footprint than plant-based proteins [55]. Additionally, fiber in raw materials can influence waste generation and resource use, further impacting environmental outcomes [56]. (2) Low humidity, since water is an expansion factor during extrusion [45,57]. (3) Cooling temperature: if it is too slow, EI may be reduced [58,59]. Another factor to consider for the EI is the viscoelastic properties of the extruded snacks. For samples with higher elastic moduli than the viscous moduli, expansion may be attributed to typical tensions, typically known as die swelling, which can occur at low temperatures and is associated with an increase in elasticity [60,61].

On the other hand, apparent viscosity refers to the relationship between the mass and volume of a substance, including the empty spaces between particles. In the food industry, BD is used to evaluate the texture and quality of products such as flour, cereals, and snacks. Low BD is associated with lighter and airier products, while high BD indicates more compact and denser food. In extruded foods, BD is a measure of quality in terms of texture, flavor, and consumer acceptance. For instance, a low BD may lead to the easy disintegration of the product, while a high BD may prove challenging to chew. Also, it may increase the porosity of the product, hence allowing the introduction of oxygen and humidity, which can accelerate lipid degradation and rancidity [62]. BD is an important feature in food design as it affects the packaging, transportation, and storage of extrudates. Lower BD is often associated with higher expansion and better texture, making the product more appealing to consumers. Studies have shown that manipulating extrusion parameters can effectively control BD, thereby optimizing the physical properties of the final product [63].

The importance of hardness, or breaking force (BF), in extruded foods lies in its role as an indicator of the quality and acceptance of the final product. An extruded food that is too difficult to chew may be undesirable for the consumer, while one that is too soft can be perceived as insipid or poorly attractive. In this sense, BF values obtained with a texturometer are used to evaluate the texture of food, allowing for the adjustment of processing parameters to improve quality [64]. It is worth noting that hardness is influenced by several factors, including raw material composition, processing conditions, and final product humidity, among others [65].

The reduction in hardness at high screw speeds and temperatures can be related to changes in the structure of the product during the extrusion process. At high speeds, pressure and temperature rise, which may favor expansion and, thus, increase porosity; consequently, these structural changes may lead to a softer product. On the other hand, high temperatures may increase the viscosity of the extruded food, making it more malleable, facilitating deformation, and reducing its hardness. Additionally, high temperatures cause the gelatinization of starch, resulting in a softer and less rigid structure [66]. Singh [67] fabricated an extruded of maize starch and chickpeas flour; the authors found that increasing the temperature and screw speed during the process reduced the hardness of the final product. Additionally, Gámez-Valdez et al. [68] obtained similar results for an extruded product composed of amaranth and maize starch.

Another parameter used to determine the quality of extruded food is the water absorption index (WAI). It is defined as the capacity of the food to absorb water and modify its physicochemical and textural properties. Extrusion is a process where mixing of ingredients, their cooking, and compression take place in a matrix to obtain a final product. In this sense, WAI plays an essential role in the quality of the final product, since it affects the texture of the extruded food [69]. In this study, it was observed that increasing the screw speed and reducing the temperature, WAI increased. This behavior can be attributed to the influence of the screw speed on the mixture and its homogenization, which facilitates the absorption of water. On the other hand, low temperatures reduce the denaturation of proteins and starch gelatinization, allowing a higher capacity to absorb water [70]. Although no statistical differences were encountered, the results suggest that, under specific conditions of temperature and screw speed, WAI may be significantly affected. The WAI measures the ability of extrudates to absorb water, which indicates their hydration properties. High WAI is desirable for products that require rehydration before consumption, such as instant noodles and breakfast cereals, but also to determine the acceptability and capacity for absorbing ambient moisture, to select the appropriate packaging. The extrusion process and the type of raw materials used can significantly affect the WAI [71]. It is recommended that future research focuses on increasing sample size and considers other factors that may affect WAI, including material composition and initial humidity [72].

Antioxidant activity, as determined by the ABTS assay, is a quantitative indicator of a substance’s capacity to neutralize free radicals [73]. In our study, it was observed that high screw speeds in combination with medium temperature (140–150 °C) resulted in higher antioxidant capacity for ABTS inhibition due to free phenols. This behavior can be explained by optimizing operational variables that favor the liberation and preservation of phenolic compounds, which exhibit antioxidant activity [74]. In addition to the independent contributions of both variables to antioxidant activity, their interaction should also be highlighted. High screw speeds favored better mixing and homogenization, which facilitated the liberation of free phenols; on the other hand, medium temperature may be sufficient to inactivate enzymes responsible of the degradation of these phenols.

In contrast, the DPPH test is more sensitive to lipophilic compounds with antioxidant activity than ABTS. The antioxidants in the formulation of the extruded functional food are primarily hydrophilic (ellagic acid and anthocyanins), while the carotenoids from the carrot bagasse are lipophilic; however, its principal carotenoid is β-carotene, which lacks hydroxyl (OH) or lactone groups, and thus, its antioxidant capacity is relatively low. DPPH measures only the transfer of electrons, while ABTS also includes H+; hence, DPPH typically exhibits lower antioxidant activity [74].

Hossain and Jayadeep [75] investigated the changes in liposoluble nutraceuticals, phenolics, and antioxidant activity of maize flour processed with 20, 25, and 30% humidity, as well as bioavailability in extruded foods at 20% humidity. Extrusion significantly reduced the content of the components and antioxidant activity. Retention of phytosterols in extruded food was higher (77–100%), followed by phenolics and flavonoids. Reducing power diminished threefold, while DPPH inhibition and total antioxidant activities were half of those exhibited in the crude product. In the bioavailable fraction, the content of stigmasterol, β-sitosterol, and flavonoids was higher in the crude fraction, while phenolics and antioxidant activity remained unchanged. In general, maize flour extruded with 20% humidity enhanced the bioavailability of most bioactive compounds in the lipidic fraction, phenolics, and antioxidants.

The exclusion of certain variables measured in this study can be justified by ensuring that only those variables with adequate adjustment to the model and high predictive capacity are considered. According to Thyashan et al. [76], including variables with low adjustment can lead to erroneous interpretations and reduce the model’s precision. In this study, optimum conditions were around 144 °C for die temperature and 207 rpm for screw speed. These values were determined by the superposition of areas, which identifies optimal operating conditions considering multiple response variables simultaneously [76]. The selection of optimum values is consistent with previous studies that demonstrated the importance of the screw speed and temperature on the quality of the extruded product. For instance, Hernández et al. [77] found that screw speed of 100–180 rpm, and a temperature of 100–140 °C were optimal for the production of extruded food with high physical and nutritional quality. The adequate precision and high determination coefficient in this study reinforce the validity of the results obtained.

Regarding the sensory tests conducted on the extrudes, a high level of acceptability was found. Notably, participants did not score Sample 8 with a 1 or 2 on the scale, and only 11.77% chose 3 (indifferent), which suggests that the product may be commercialized in terms of its sensorial and organoleptic properties [78]. The written descriptions mentioned that the least liked aspect of the product was the “acidity” flavor at the end; nonetheless, the crunchy and expanded texture, as well as the initial flavor, were factors that were highly accepted [79]. These findings are consistent with previous studies that have demonstrated that texture and initial flavor are key factors for the acceptance of food [80]. The results of this study indicate that Sample 8 exhibits high sensorial acceptance, which may lead to successful commercialization. The combination of crunchy and expanded texture, along with the likable initial flavor, contributes significantly to consumer acceptance. Sample 8 was one of those that presented the highest antioxidant capacity, a high EI, a low hardness (compared to the other samples), a high WAI, and a low WSI. All of this may have contributed to its improved acceptability. The optimization of extrusion conditions for an extruded food enriched with mango by-products demonstrated that higher antioxidant capacity, expansion index (EI), and water absorption index (WAI), along with lower hardness and water solubility index (WSI), can significantly enhance the quality and acceptability of the final product [81]. Additionally, a comparative study on antioxidant capacity highlighted the importance of polyphenol and flavonoid content in improving the nutritional and sensory properties of food matrices [82].

Several key characteristics can be highlighted in the nutritional content of the final extrudes. The protein content was 8.9 g/100 g of product, which can contribute to the maintenance and repair of corporal tissues [83]. Also, the product can be considered as low in fat according to the Official Mexican Norms NOM-051-SCFI/SSA1-2010 (a product can be regarded as “low in fat” when it contains no more than 3 g of fat/100 g or 100 mL of product), since the fat content was of 2 g/100 g of product; this could assist in reducing the risk of suffering from cardiovascular diseases, along with following a healthy lifestyle [84,85]. In addition, the product contains a moderate amount of fiber, which may contribute to moderate glycemic index properties. Carbohydrates that can produce energy account for 73.5 g, while total reducing sugars (7.0 g) maintain a moderate level. These parameters may become favorable for controlling blood glucose and preventing non-alcoholic steatohepatitis when combined with a healthy lifestyle and dietary patterns suitable for the population [19,86].

Interestingly, the energetic content of 348.3 kcal/100 g of product can be considered adequate for energy provision without exceeding daily recommendations, which is essential for maintaining corporal weight [87]. The product exhibits an energetic density inferior to commercial snacks, which typically range from 500 to 600 kcal, due to the high content of fats and oils added during the frying process. Finally, a sodium content of 83.0 mg can be considered low and beneficial for maintaining healthy arterial pressure [88]. Overall, extruded snacks offer a healthy nutritional profile, featuring a good balance of protein, dietary fiber, and carbohydrates, along with a low content of fat and sodium. These characteristics can contribute to the promotion of health and the prevention of diseases.

The snacks prepared in this study were supplemented with ellagic acid, a polyphenol known for its multiple health benefits, including antioxidant, anticarcinogenic, and anti-inflammatory activities [24,27]. The loss of 50.5% of ellagic acid during the extrusion process was significant. However, the residual amount of 89 mg/10 g is considerable and can contribute to the health benefits of the final product [89]. Previous studies have demonstrated that ellagic acid may prevent certain types of cancer by inducing apoptosis of cancer cells and protecting the DNA from oxidative stress [90]. Additionally, anti-inflammatory properties may reduce the risk of developing chronic illnesses, such as arthritis and cardiovascular diseases [91]. Furthermore, the antioxidant capacity of ellagic acid is notable, as it can neutralize free radicals and reduce oxidative stress in the body, as well as neuronal damage [26,89].

The stability of ellagic acid depends on the procedure by which it is incorporated into the food. For example, Marić et al. [92] found that ellagic acid stability in raspberry extruded products is not significantly affected by temperature and screw speed during extrusion. The authors successfully retain the content of ellagic acid above 60% under process conditions of up to 200 °C, suggesting that adjusting this parameter may enhance the retention of the compound [92]. Although a loss of 50.5% of ellagic acid occurred during the extrusion procedure in this study, the residual content remained significant regarding its contribution to health benefits, as intake above 28 mg daily has been shown to provide metabolic benefits [93]. Thus, optimizing the operational parameters of extrusion may improve the retention of ellagic acid, thereby maximizing its beneficial properties in the final product.

The potential limitations of this study include the use of an untrained sensory panel that may introduce variability and subjectivity in sensory evaluation results. Variations in the chemical composition of nixtamalized corn and carrot bagasse may affect the final food composition. In addition, the unique characteristics of these raw materials may not be similar to those available in other regions or from other suppliers.

## 5. Conclusions

A functional snack was prepared from agroindustry residues, composed of bagasse from carrots, blue maize nixtamalized flour, and ellagic acid, through extrusion. This grass-based food can be considered an alternative in the development of functional snacks while also contributing to the utilization of waste produced in industry, thereby adding value and promoting both ecological and human health perspectives.

From a functional perspective, the optimal conditions of temperature and screw speed of the extruder modulate the antioxidant capacity of the snacks. The experimental design should avoid extreme values, as a very low optimal temperature may hinder the liberation of bioactive compounds in the food matrix, making their absorption difficult and reducing their bioavailability. In addition, this food may serve as a nutritional option for populations seeking healthy alternatives to conventional snacks due to its low sugar and lipid content, as well as the incorporation of natural antioxidants such as ellagic acid, anthocyanins from blue maize, and carotenes from carrot bagasse. The optimal processing conditions in our study were 144 °C DT and 207 rpm screw speed. Based on our findings, we can consider that a high expansion index, high BD, low breaking force, high antioxidant capacity, high WAI, and low WSI are ideal for good acceptability of the extruded product. Likewise, moderate temperatures and screw speeds can result in an optimal combination of all these characteristics. For this, the second-generation snacks described in this research may assist in the treatment and prevention of pathologies such as type 2 diabetes mellitus, insulin resistance, dyslipidemias, metabolic syndrome, atherosclerosis, and fatty liver disease associated with metabolic malfunction, all this integrated with a healthy lifestyle with regular exercise, and adequate dietary patrons according to everyone.

## Figures and Tables

**Table 1:** Experimental design used for formulation extrusion.

	Independent Variables			
	Coded		Actual	
Treatment	*X*_1_	*X*_2_	DT (°C)	SS (rpm)
1	+1.414	0	170	145
2	0	0	145	145
3	−1	+1	127.322	212.175
4	+1	−1	162.678	77.8249
5	−1.414	0	120	145
6	0	+1.414	145	240
7	0	0	145	145
8	0	0	145	145
9	+1	+1	162.678	212.175
10	−1	−1	127.322	77.8249
11	0	0	145	145
12	0	0	145	145
13	0	−1.414	145	50

Note: DT: die temperature (°C); SS: screw speed (rpm).

**Table 4:** Nutritional content per 100 g of final extruded produced at 144 °C and 207 rpm of screw speed.

Determination	Result	Nutrient Reference Values (NRVs)
Humidity	6.5 g	N/A
Ashes	2.4 g	N/A
Proteins	8.9 g	17.8%
Total fat	2.0 g	2.9%
Saturated fat	2.0 g	10%
Unsaturated fat	0.0 g	N/A
Dietary fiber	6.6 g	26.4%
Total carbohydrates	73.5 g	24.5%
Total reducing sugars	7.0 g	14%
Energetic content	348.3 kcal/1457.3 kJ	17.4%
Sodium	83.0 mg	3.6%

N/A: Not Applicable. The numerical values presented in the table are the results of the average of the quantifications performed in duplicate.

## Data Availability

The original contributions presented in this study are included in the article. Further inquiries can be directed to the corresponding authors.
